# Civil Servants and Non-Western Migrants’ Perceptions on Pathways to Health Care in Serbia—A Grounded Theory, Multi-Perspective Study

**DOI:** 10.3390/ijerph181910247

**Published:** 2021-09-29

**Authors:** Sofie Buch Mejsner, Maria Kristiansen, Leena Eklund Karlsson

**Affiliations:** 1 Unit for Health Promotion Research, University of Southern Denmark, Degnevej 14, 6705 Esbjerg, Denmark; leklund@health.sdu.dk; 2 Department of Public Health, Center for Healthy Aging, University of Copenhagen, Øster Farimagsgade 5, 1353 Copenhagen, Denmark; makk@sund.ku.dk

**Keywords:** informal patient payments, out-of-pocket-payments, health systems governance, Serbia, migrants and civil servants

## Abstract

(1) Background: Informal patient payments continue to persist in the Serbian health care system, exposing vulnerable groups to private spending on health care. Migrants may in particular be subject to such payments, as they often experience barriers in access to health care. Little is known about migrants paying informally to access health care in Serbia. The study aims to explore pathways of accessing health care, including the role of informal patient payments, from the perspectives of civil servants and non-western migrants in Serbia. (2) Methods: Respondents (*n* = 8 civil servants and *n* = 6 migrants) were recruited in Belgrade in 2018, where semi-structured interviews were conducted. The interviews were analysed applying the grounded theory methodological steps. (3) Results: Data reveal different pathways to navigate the Serbian health care system, and ultimately whether paying informally occurs. Migrants appear less prone to paying informally and receive the same or better-quality health care. Locals experience the need to pay informal patient payments, quasi-formal payments and to bring medicine, materials or equipment when in health facilities. (4) Conclusions: Paying informally or using private care in Serbia appear to have become common. Despite a comprehensive health insurance coverage, high levels of out-of-pocket payments show barriers in accessing health care. It is highly important to not confuse the cultural beliefs with forced spending on health care and such private spending should be reduced to not push people into poverty.

## 1. Introduction

In health care, governance is the steering and rulemaking related function that is carried out by decision makers when attempting to reach national health policy objectives and improve the well-being of populations [1]. Governance concerns the interaction between governments and other social organizations, the decision-making process, and the inclusion and interaction with citizens [2]. Defining health system governance includes the principles of transparency and equity in access to health care [3,4,5]. One of the main pillars of the European Union is in particular timely access to affordable health care of good quality [6]. However, waiting times and financial barriers to access health care are evident across Europe and especially among vulnerable groups such as migrants and refugees [7,8,9,10,11,12,13,14,15,16].

A substantial proportion of refugees and migrants originate from countries where health care systems are weak and characterized by low quality and poor access to services [17]. Many migrants face hardships through their journey, leading to serious illnesses such as post-traumatic stress disorder, mood and anxiety disorders, panic attacks or vaccine-preventable diseases, for example, hepatitis, tuberculosis, and measles [12,18,19,20]. Becoming a part of European societies, the migrants will be further susceptible to a variety of non-communicable diseases [19,20,21]. European Union member states are, therefore, faced with pressing needs to address these public health consequences and the challenges to adequately address migrants’ health care needs [13,18,19,20,21,22].

The Western Balkan countries were affected by the influx of migrants in 2015 [23,24,25]. In particular, Serbia was affected, as the country was part of the ‘Western Balkan Route’ [24,25,26]. Approximately 100,000 migrants transited Serbia, but because of border closures in March 2016, up to 7700 migrants remain stranded in the country [26,27]. Today the events in Africa and the Middle East continue to raise migration trends, with approximately 126,000 in 2019, 94,405 in 2020 and 16,217 in the first part of 2021 arriving via the Mediterranean countries [28].

In a humanitarian crisis, like the influx of migrants in Western Balkan, it is vital that health care systems function and are able to cope with the increased pressure of migration flows [23,29]. Migrants need human rights protection and health care from local clinics/hospitals since vulnerability increases during migration, and since they are exposed to poor travel conditions, injuries, traumatic events, and lacking access to health care [17,26,30]. Most Western Balkan countries are reluctant to provide health care services to some migrants such as irregular migrants or asylum seekers [26].

The Serbian health care system is characterized by universal health coverage that is both publicly financed and provided. The National Health Insurance Fund manages the contributions made to the fund. Sometimes co-payments are required for selected services or private insurance can be obtained to enhance consumer choices [31,32]. Reforms were recently implemented to improve access to the health care system and to reduce inequalities in health [33]. Being a country with economic, social, and demographic difficulties, the Serbian health care system suffers from underfunding [24,34,35,36]. One of the symptoms is the informal patient payments that have shown to be common in Serbia and other Western Balkan countries [37,38,39]. These payments may be in the form of presents (e.g., chocolates, drinks), small tips or large sums of cash [40] and are perceived as being outside the official system. Research emphasizes that informal patient payments are symptoms of poor management, underfunding, political weakness, lack of accountability and deficits in rule of law—therefore, poor governance [41,42,43]. Other studies reveal that informal patient payments compensate for in example, poor salary or lacking availability of drugs [37,44,45,46,47,48,49]. Such health systems are often characterized by inequality, injustice, and denial of basic rights [1]. Some evidence suggests that migrants in the Western Balkans may pay informally more often than other population groups [47,50].

In the beginning of the 2015 migration crisis to Europe, Serbia was viewed as a transit country and mainly emergency care services were being provided to migrants [51]. Closing the Balkan Route in 2016 stranded people in Serbia and thus the country’s health care system needed to respond to the changing circumstances [23,51]. Therefore, a strategy and action plan were implemented to deal with the increased number of migrants, where several initiatives were taken towards better quality care and access to services for migrants [51].

The large influx of migrants was not predicted and financing of services for this group was difficult. Migrants are generally covered by the national compulsory health insurance scheme and may receive services defined in this scheme, some with additional co-payments [32]. They access primary health care services at the asylum centres or can be referred to specialist care services at the local hospitals or clinics. Intercultural mediators and sometimes health care professional assist the migrants when leaving the asylum centres to access specialist care [32]. Primary care services have been financed by NGOs, whereas public secondary and tertiary care was not reimbursed, forcing hospitals to cover these costs [51]. The cost, therefore, went to the public health care institutions, which were already under constraints [33,35,36,52,53,54].

Until now, studies about access to health care have primarily focused on Roma People and Bosnian immigrants. Little literature is available on access to health care of other vulnerable migrants, such as the recent influx of non-western asylum seekers and refugees in Western Balkan countries [9,26,55,56] and whether they pay informally to access health care.

In Serbia, the intercultural mediators and information workers from the NGOs were often highly important when non-western migrants needed to navigate the public health systems. They thus had valuable knowledge about non-western migrants’ access and experiences with the public health care systems. For simplicity, non-western migrants will be mentioned as migrants in the study, and intercultural mediators and information workers as civil servants. The present study therefore aims, to explore pathways of accessing health care, including the role of informal patient payments, from the perspectives of civil servants and migrants in Serbia.

## 2. Materials and Methods

Grounded in empirical evidence, we sought to uncover the processes inherent to paying informally in Serbia. We adapted a grounded theory method [57] in our study, with a multi-perspective approach. Such an approach allowed us to uncover multiple understandings of the topic in question, from the perspective of both the migrants and those working in the field of migration.

In the 1960s, grounded theory was originally developed by Glaser and Strauss [58]. Grounded theory was intended to be a new approach to qualitative research serving to explain the studied phenomenon as opposed to, for example, explore or describe the phenomena [59]. Others have sought to further develop the method, such as Bowers and Schatzman [60], Charmaz [57] Clarke [61], known as second-generation grounded theorists. Thus, there are different methodological genres (e.g., traditional, evolved, constructivist) to be used in grounded theory research, with each seen as an extension of the original work by Glaser and Strauss [59,62]. This study identifies with Charmaz’s genre of *constructivist* grounded theory because we believe that data are co-constructed by the researcher through interaction with the respondents. Data collection and analysis is coloured by the researcher’s perspectives, values and interactions [57]. Data are thus not discovered without preconceived ideas about the concepts in question. The pre-existing knowledge was used to enhance the theoretical sensitivity, without forcing conclusions on the collected data. Using Charmaz’s genre, we were thus provided with practical steps in data collection and analysis [57,59].

### 2.1. Qualitative Interviews

#### 2.1.1. Ethical Approvals and Informed Consent

An ethical approval was given by the Faculty of Medicine at the University of Belgrade (Number: 2650/VI-12). In Denmark an ethical approval was received from the University of Southern Denmark (SDU) (Number: 18/33066 CEAN) and the South Regional Committee on Health Research Ethics (Number: S-20182000-84). All participants signed an informed consent form before conducting the interviews. We further followed the SDU Data Protection Guidelines, based on GDPR, ensuring data confidentiality, and safeguarding of data files.

#### 2.1.2. Sampling and Data Collection

In August and September 2018, qualitative interviews were conducted with civil servants and migrants living in asylum centres. These two groups of interviewees made it possible to ensure the consistency and credibility of the findings. The civil servant interviewees worked in the field with migrants or were involved in an organization dealing with migration. They included information officers and intercultural mediators were working with the local NGOs and were aged 25–53. Six were Serbian nationals and two were from other European countries. The migrant interviewees were asylum seekers or intended asylum seekers, residing in an asylum centre near Belgrade. They were aged 19–62 years, while two were from Iran and four from Afghanistan. The migrant interviewees were invited to participate by staff at the centre. To ensure informed consents, migrants were asked orally and were provided with written materials on the purpose of the study.

The University of Belgrade, Serbian Red Cross, the Danish Refugee Council, Doctors Without Borders, two Belgrade-based NGOs, and the local WHO unit for Serbia assisted during the spring of 2018 in finding and recruiting interview respondents. Inclusion criteria were migrants from an asylum centre or civil servants in the field of migration. The University of Belgrade also assisted in obtaining access to an asylum centre near Belgrade, to interview migrants. Respondents (eight civil servants and six migrants; eight men, six women) were recruited in Belgrade with a purposive sampling method. Snowballing sampling was also needed to recruit other respondents from among acquaintances.

Since the migrant populations are highly diverse, only asylum seekers or intended asylum seekers were included, to generate a meaningful analysis. Thus, we only included non-western migrants and not the internally displaced or regional Western Balkan migrants. The included migrants had lived in an asylum centre for a period of three months to three years. All interviews were conducted in English, using an interview guide with four overall questions. The interview guide thus provided a skeleton for all interviews. The interviews with migrants were assisted by local researcher from the University of Belgrade and an intercultural mediator. The intercultural mediators spoke their local languages (Persian, Dari, Farsi) and knew the migrants in advance. Two intercultural mediators assisted, and the interviews were, therefore, organized in terms of migrants’ languages. All civil servants spoke English fluently. All interviews were recorded on a dictaphone and lasted from 45 min to 2 h.

#### 2.1.3. Data Analysis

The interviews were analysed applying the grounded theory methodological steps [57,59] by first condensing the text into codes and categories, and in parallel collecting new data to evolve new categories. The initial coding was completed by identifying important words or sections of the interview transcripts and labelling them accordingly. The data was then divided into overall categories, from where the most significant codes were selected, synthesized, and explained, also called focused coding [57,63]. A fundamental part of the grounded theory method is concurrent data collection and analysis, as well as the constant comparative analysis [57,59,63]. Concurrent data collection and analysis was conducted by listening to the interview tapes immediately after the interview, including writing down the initial codes that were then used in and adapted for the next interviews. During the analysis of data, the constant comparative analysis included comparing codes and categories with each other. Memos were used to write down discoveries, questions raised in the researcher’s mind, and ideas emerged during the analysis. These memos were used later to conceptualize the data. Respondents were recruited during the collection of data, to find those that would be suitable to explain these new ideas and questions that arise. This theoretical sampling made it possible to elaborate meaning, discover variations and define gaps among the categories. Memos were used from the early stages to the completion of a concepts, recording the thoughts and ideas about the data. These steps were an iterative process that continued until saturation, meaning that no new categories emerged. NVivo was a used to assist in the analysis of the data.

#### 2.1.4. Theoretical Integration

Advanced coding is critical to theoretical integration and is a core part of the grounded theory method. It is a comprehensive method that includes storytelling and theoretical coding [57,59]. Thus, the relationships between the concepts that make up the theory are explicated. Memos and diagrams were used to intellectually raise the data by analysing, developing and scrutinizing the codes in an advanced stage [59].

Creating the models of pathways to paying informally was a process of abstraction, reflecting on the concepts/categories and their relationship and a re-confirmation from the collected data. Negative cases from the results, that would contradict the initial models, were used to revise them. Furthermore, the already existing theory about Institutional Asymmetry [64,65] catalysed the development of the models of pathways. Thus, the theory’s distinguishing between formal institutions, with codified laws and regulations, and informal institutions with socially shared (unwritten) rules inspired the models. The higher the asymmetry between the institutions, the higher is the likelihood for informal patient payments. Institutional asymmetry, therefore, occurs when, for example, informal institutions take over the formal ones that are performing imperfectly [64,65,66]. In that sense, the theory also inspired the depicturing of the complex interaction occurring between formal and informal institutions when paying informally for health care.

## 3. Results

**Figure 1:**
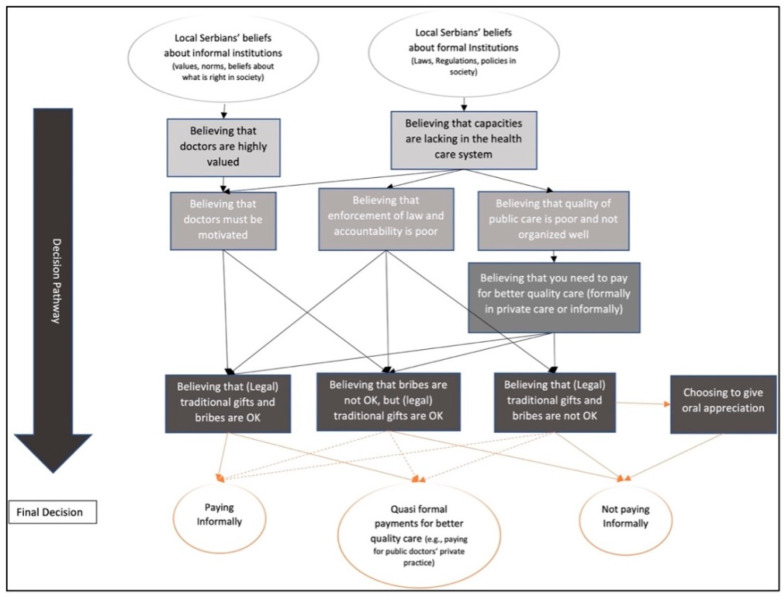
Illustration of potential pathways to informal patient payments perceived by civil servants.

**Figure 2:**
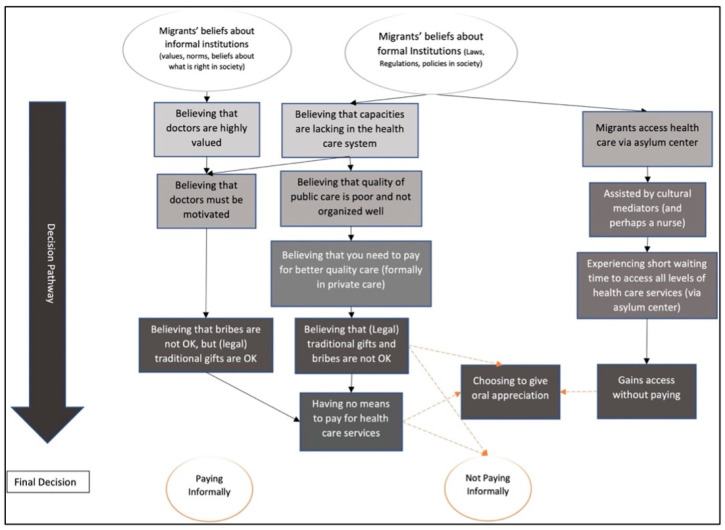
Illustration of potential pathways to informal patient payments perceived by migrants.

The data revealed that migrants and local residents had different pathways when navigating the Serbian health care system, and ultimately whether paying informally. Generally, informal patient payments were perceived as unacceptable by all respondents and none of them had given such payments in Serbia. However, some distinguished the illegality of paying informally from the practice of giving gifts to motivate doctors. Migrants mostly reported narratives about their own experiences, whereas civil servants gave their views on the migrants’ experiences and the practices they believed occurred in the Serbian health care system. The results are illustrated in Figure 1 and Figure 2.

### 3.1. Civil Servants’ and Migrants’ Perceptions of Informal Patient Payments

Giving illegitimate gifts and money to health care providers is a practice that remains present in Serbia. All local civil servants expressed that there was an illegal and unacceptable aspect of giving gifts and money to health care providers.

*I mean it’s for me I don’t approve it at all. Not before, not after the treatment (…) I think people are pissed off but I think all of them will do that if needed.* (Civil Servant 2)

The payments were often rooted in the lack of resources of the health care system. Often informal patient payments would compensate for the poor working conditions, the low salary of doctors or the lacking equipment or drugs in the institutions. Generally, there was an acceptance that bringing goods, like sheets, medicine, equipment, to the hospitals was common and acceptable (due to the lack of resources). For some, gift giving was also acceptable in a traditional view and the respondents expressed that it should be allowed in some form. Most of the civil servants believed that the reason for traditional gift giving between health care professionals and patients was because of the high value placed in doctors. Doctors thus have a very important duty to carry out and patients may perceive this as almost ‘godlike’.

*So you have some layers of society who are more powerful than others. Doctors now have power over life and death, like the clergy did before. And just a way to honour this power. So, it is something like a tradition basically.* (Civil Servant 1)

Doctors in Serbia, however, also work under miserable conditions, such as receiving a poor salary, working long hours, lacking equipment or receiving poor skills development. Thus, informal patient payments were viewed as a legitimate way to compensate doctors (see Figure 1 and Figure 2). Generally, respondents believed it was acceptable to motivate or pay respect to doctors for doing a good job or for working in the public health care system, despite the poor working conditions. Some of the respondents disputed this, saying that they, as civil servants, were not rewarded for their job so why should doctors be. This latter group were typically completely against any form of gift or money sharing in health care. They believed there was no such thing as a gratitude gift to health care providers.

*This is one of the things where people are talking about cultural you know; I really don’t believe in that because the thing is that this is just a pure bribe.* (Civil Servant 3)

Most migrants were also familiar with the concept of giving gifts and money to health care providers, mostly from their country of origin and in situations like giving birth or having a serious operation. Typically, they would describe it as a gratitude gift, showing gratitude for the positive experience. They would further have done similar to Serbian health care providers, if they had the means to do so.

*(…) they gave you drugs you know they help you when they take care of your problems, they cure you, you know, the next time you go there you say thanks to them.* (Migrant 4)

Migrants and civil servants, however, all agreed that migrants did not have the means to either give gifts or cash to health care providers in Serbia. They were in some ways protected from this when being assisted by intercultural mediators and nurses.

#### Informal Patient Payments as a Regular Practice

Several of the civil servants believed that paying informally was decreasing in society. All of the respondents seemed to be aware of the unlawful practice of informal patient payments. Most of them believed that it occurred primarily when seeking care from specialists, typically when needing surgery or maternity care. The respondents especially referred to maternity care as an example for illegitimate and legitimate bribes. The former as an informal patient payment and the latter as a ‘quasi-formal payment’ (see Figure 1 and Figure 2). Several civil servants had themselves or with relatives experienced a form of ‘legal bribe’ in maternity care. They distinguished this from informal patient payments, since it was reported to be a well-known practice that most people adopted to ensure better quality care. Thus, patients would pay a doctor, at his own private practice, to take care of them when giving birth in a public hospital. If patients chose not to pay extra for a private doctor, the respondents explained that these patients were at risk of being treated poorly. The fear of poor treatment when giving birth led patients to pay the extra money, the respondents reported.

*(…) When you go to a doctor in a hospital (…) it’s tradition to also go to a private practice of this doctor, and me and my ex-wife we just never did that. (…) nobody would ask her anything like ‘are you feeling okay’. All the other patients would be constantly visited by their doctors (…) who they hired privately in their private clinics. My ex-wife’s doctor would never come and check up on her or anything like she just gave birth, and after that it was done.* (Civil Servant 3)

Thus, it was a common perception that if a patient refused to ‘buy’ a doctor, the service level to the women giving birth would be significantly poorer (e.g., being ignored, being spoken improper to, or being denied treats, food, water, or the like). Most patients were not willing to take that risk and it was, therefore, believed to have become a systematic practice to pay informally. According to the respondents, this practice had become so regular that patients were used to it and expected it to occur.

### 3.2. Multiperspective Perceptions on Pathways to Informal Patient Payments

The respondents mentioned repeatedly that lack of resources as one of the main reasons for the poor functioning health care system. The poor functioning of the health care system was thus perceived by both civil servants and migrant as the triggering element to the pathway of paying informally. Another element was the excessive value that patients placed on doctors, believing in their ability to cure their illness better than any other professional, and thus hoping for restoration in health. Those beliefs were crucial when choosing to pay informally (Figure 1 and Figure 2).

The data revealed different pathways for migrants and local residents when accessing health care services and their reasoning of paying informally.

#### 3.2.1. Civil Servants’ Perceptions on Pathways to Informal Patient Payments

Civil servants all agreed that the Serbian health care system was under-funded, but some were also sceptical about the financial support from EU or other international organizations. Thus, if the Serbian health system was expected to function without international assistance, there would be a need to reform the entire system. Respondents believed that willingness to change and funding were needed within health care professionals, local residents and decision makers.

*At the moment it looks unfixable like really there is some kind of a disease in the health care system. The problem is with the not so huge budget, and more funds is not going to solve this. Of course, you will motivate doctors and people to work better if they have better salaries and better conditions but that’s like a second thing. The first thing is that you need to do something else to improve it.* (Civil Servant 6)

The resource drained health system had several consequences for its functioning. One being the brain drain of Serbian doctors to European countries and, another, the overburdened health care system. One respondent called it “a permanent fatigue” (Civil Servant 3) of doctors and claimed that migrants coming to Serbia may be the ones to “tip them (read doctors) over the edge” (Civil Servant 3). The meaning of this was that carelessness and other informal or poor behaviour would likely increase if no actions were taken to alleviate the migrants’ accessing health care.

Civil servants described several issues of poor quality or organization of services, such as long waiting lists, the ambulances being late, speaking to patients in incomprehensible or rude language, denying treatment of the elderly (merely because of age) or denying migrants the care that they need. The respondents believed that the health care systems’ poor resources made money or gifts acceptable to motivate health professionals. They thus argued that public doctors could, for example, have chosen to work in a private clinic with a much larger salary. Instead, they chose to stay in public institutions and supporting these, which should be rewarded.

Poor quality and organization of the public health care system led people to private health care services. There was thus a general narrative that if people had money, they would choose private institutions. Stated implicitly, those who did not have the money to pay for their own medical treatments in a private clinic, would have to accept the level of treatment that was given to them in public institutions. There was a general idea that private institutions for instance, were cleaner, had better services and facilities. Even though it was expensive to pay a private clinic, some would never go to the public health care institutions but would always choose private care.

*If you have money, that is. You would never really go to the public institutions. You would choose the private because they are just better.* (Civil Servant 1)

In some areas of public health care, such as the long waiting lists, access to services was limited. Civil servants believed that if paying yourself to have, for example, surgery in a private clinic, it could be done immediately instead of waiting for almost a year.

#### 3.2.2. Migrants’ Perceptions on Pathways to Informal Patient Payments

Migrants were aware of the issues of poor quality or organization of services. They described experiences of doctors prescribing the cheapest medicine regardless of the side-effects, general practitioners not sending very ill children further to the hospital or being spoken to rudely. However, the interviewed migrants came from poor conditions in either their home country or countries surrounding Serbia. They were, therefore, satisfied with the access to health care services that they received when compared to the services in their home countries. For some selected services, migrants would have to wait to be treated since they were incapable of paying for private care. Otherwise, they generally did not experience poor health care resources to the same extend as other local residents did, mainly as they accessed health care through different institutions. Some locals would become upset about migrants’ quicker health care access, but usually it did not raise problems. Migrants were further aware of the differences between private and public institutions and would typically have had the same experiences in their native countries.

*(…) if I have good money for sure I would go to private and (receive) good service about clinic, hospitals, staff, materials, anything.* (Migrant 2)

The first entry point for the migrants was the available doctor in the asylum centre. If needing further medical care, they were sent to the local hospitals. Here, they would typically (but not for all services) receive an appointment quite quickly, contrary some local residents. They would thus bypass waiting lists.

Migrants were further supported by an intercultural mediator and sometimes a nurse. Generally, the intercultural mediators were crucial for the migrants in accessing health care since there was a high degree of trust between them. Migrants would call the intercultural mediators after working-hours or during the night if they had emergencies, and the intercultural mediators would assist them. By having a broad understanding of the migrants’ background and being always available, intercultural mediators created and maintained a high degree of trust. Being assisted by intercultural mediators and nurses made it nearly impossible for the migrants to give informal patient payments to the health care staff, due to the high degree of control. It also made access to services easier since there was a higher degree of organization. Typically, the doctors would have timeslots for migrants that could be booked within a few days. The access to health care was thus very organized, making it easier for the migrants to receive the services needed.

The differences in access to health care between local residents and migrants was viewed as a consequence of the rapid influx of migrants to the Western Balkan countries. Thus, other migrants residing in Serbia still faced obstacles in accessing health care services such as language barriers, cultural understanding and utilization of services.

## 4. Discussion

The present study found different pathways to paying informal patient payments of migrants and local residents. Different pathways to and through the health care system were found, resulting in different outcomes when it comes to informal patient payments. The respondents reported many of the same issues such as lacking resources in the health care system and the high value that patients placed on doctors in Serbia. Such high value was perceived as one of the main reasons for the pervasiveness of informal patient payments, believing that doctors needed motivation for working under miserable conditions or to receive gratitude for the service given to patients. Several respondents mentioned that it was a cultural phenomenon to view doctors as almost ‘godlike’ which correspond to other literature, finding patient-provider relationships to vary according to culture [67,68,69]. Health care systems and governance systems varies and are thus shaped by the social context and relies heavily on the inclusiveness and responsiveness to culture, social justice and human rights [2]. In some cultures, doctors may, therefore, have more authority in the decision-making process than patients, and patients thrive in a paternalistic relationship with them [70].

The lack of resources in the Serbian health care system was generally understood to cause poor access to health services and poor quality of health services. Thus, respondents had higher acceptance of giving informal patient payments or quasi formal payments. It seemed that they would rather use private health care services, despite being almost entirely based on direct out-of-pocket payments and unregulated by the state. The social health insurance coverage is 98% [32] and thus there appears to be staggering gaps in the utilization of services.

The issue of constrained resources in the Serbian health system is well-known [2,32]. The consequences may be the inequities in access to Serbian health care that is observed for particularly vulnerable groups, such as Roma and the people uninsured from the National Health Insurance Fund [32]. High levels of reported unmet needs among, for instance, women, the elderly, lower educated and those with lower income are seen in Serbia, together with high levels of private out-of-pocket payments [71]. Correspondingly, the most reported unmet need reported by Serbians were financial reasons, followed by waiting times and distance or transportation difficulties. Such challenges in health care systems are not uncommon. A 2019 study from WHO [72] showed that nearly half of the worlds’ population cannot obtain the necessary health care services. They therefore pay out of pocket, leaving them without financial protection while being pushed into poverty. Several studies on informal patient payments [43,73,74,75] have similarly found that those with money are more likely to pay informally. This may correspond with the findings on unmet needs in Serbia, while those who access care are those that are able to pay. The high unmet needs are, therefore, largely present to the most vulnerable groups.

The findings from the present study, however, illustrate another dimension of migrants accessing health care. The respondents all agreed that newly arrived migrants in Serbia were given easy access to most services through intercultural mediators, quick access to services and the presence of primary care doctors in the respective camps. Generally, the compulsory health insurance scheme covers migrants, Roma and unemployed from the state budget [32]. They are thus covered officially, but barriers exist for some when accessing health care. In particular, research shows that any cultural insensitive or misunderstood encounters with the health care system can undermine trust in the system and make migrants reluctant to utilize services [76,77]. The newly arrived migrants in Serbia were, however, given greater attention by authorities. In this regard, the WHO Country Office in Serbia supported the Ministry of Health in Serbia in developing and implementing various interventions [51]. The plan followed the *‘Strategy and action plan for refugee and migrant health in the WHO European Region’* [78] where priorities and strategic areas addressed specifically the public health and health system challenges related to migration. This plan appears to have made a difference in the access to health care between these newly arrived migrants and other vulnerable groups (e.g., other migrants) in Serbia, who otherwise reported high unmet needs and barriers in accessing health care. The initiatives implemented in the current humanitarian situation was the use of intercultural mediators when accessing health care services; establishing a framework for collaboration between relevant public and private sectors; and training of professionals in the provision of mental health, psychosocial support and communicable disease prevention [51]. The lack of access to health care services is a highly important issue, as it may cause migrants to seek alternative health-seeking strategies that undermines the health care systems [79]. The aforementioned initiatives may therefore be taken into consideration when addressing the barriers to access health care services of other vulnerable groups in Serbia.

### 4.1. Addressing the High Level of Out-of-Pocket Payments and Informal Patient Payments

The Serbian health system faces challenges such as migration, fiscal sustainability, and an aging population [32,80,81,82]. However, Serbia has increasingly overcome several obstacles in health care since the 1990s break-up of Yugoslavia. Increases in the welfare of citizens, the purchasing power and living standards have been seen over the past years [32,83]. Such factors are also indirectly important for a well-functioning health system [78,84]. In Serbia, health system reforms focused on improving the infrastructure and technology and implementing health information systems [32]. Although the system also faces challenges in several areas, e.g., a shortage of human resources, difficulties with the newly build health information systems, poor health financing and difficulties in investing in technologies [32].

Poor-performing health systems, measured in the financial protection of patients, typically have higher levels of out-of-pocket payments [85]. As part of the direct out-of-pocket payments, informal patient payments also become more frequent. Such unregulated direct payments to health care providers are one of the main barriers to access health services and may push people into poverty, as health expenses absorb the household’s financial resources [85]. In the last decade, public sources of funding for health have decreased while consequently increasing the private out-of-pocket payments in Serbia. The private out-of-pocket payments levelled at approximately 40% of total health expenditure over the past years [86]. These include also informal patient payments and correspond to the findings of the present study, regarding the commonness in using private care or paying informally for services.

Some respondents believed it was acceptable to give gratitude gifts, whereas others were completely opposed, calling it corruption. This resembles the present duplicitous aspects when attempting to combat informal patient payments in health care. There are various beliefs about whether payments are a fee-for-service or out of gratitude, whether they are forced or voluntary and what it constitutes when they are paid in money or given gifts (e.g., beverages, flowers, chocolate). Beliefs about money or gifts being given out of gratitude versus out of corruption is discussed in numerous literature on informal patient payments [37,87,88]. These issues are no exemption in Serbia [37,48,52]. In an attempt to combat the illegality of informal patient payments, in 2019 Serbia adopted the new Health Protection Act (Zakon o zdravstvenoj zaštiti “Official Gazette of RS”, No. 25/2019) and Health Insurance Act (Zakon o zdravstvenom osiguranju “Official Gazette of RS”, no. 25/2019), which legalizes gifts worth up to 5% of the average monthly net salary in Serbia to health professionals. Legalizing gifts may be viewed as a de-criminalization of citizens, but also greatly increases the likelihood of direct out-of-pocket payments, which can have harmful economic effects on private households.

The legalization of gifts to health care providers may be based on an acceptance that there are national-cultural aspects of doing so. Thus, banning the practice may lead to further informal and illegitimate behaviour, as people would most likely not end the practice. Legislators may, therefore, have reversed the (to legislators) illegitimate practice to be a legitimate one. In this regard the institutional asymmetry perspective [64,65] distinguishes between informal (socially shared rules) and formal institutions (codified laws and regulation) [66]. When formal and informal institutions are in alignment and consequently state morality is in symmetry with individual morality, then the illegitimate practices (as perceived by the state) will be largely absent. Thus, the socially shared norms and values are aligned with the formal rules [65]. In that regard, Serbian authorities may attempt to acknowledge the cultural elements that are present when paying informally and consequently aim at formalizing the practice. Attempting to align state and individual morality may be important when talking about e.g., trust in government and the rule of law and thereby also the health system governance [87,89,90]. However, the high level of out-of-pocket payments seen in Serbia poses the question of whether patients voluntarily choose to pay privately or because there is a need to. Caution in shifting the responsibility of financing health care services to patients is in this regard highly important.

When discussing the legitimacy of informal patient payments, the institutional asymmetry theory poses that it is only when the socially shared (informal) rules and the formal rules deem a behaviour illegitimate, that it may be classified as criminal behaviour (e.g., human and drug trafficking). Whether informal patient payments are criminal behaviour or not may vary between people’s perceptions. In the present study there were various opinions about gift giving between patients and health professionals. Nearly all respondents believed that bribery was not acceptable, however, it was necessary at some point. The criminalization of the phenomena will depend on the socially acceptability by citizens. Such acceptance may change with generations and therefore it may have been acceptable but is not anymore [64].

### 4.2. Considerations about Methodology

Qualitative research methodology can rarely be generalized due to small sample sizes [91]. In the present study, we recruited 14 participants and reached saturation of data, though generalizability is not present. The civil servants were selected in a purposeful manner, to conduct the theoretical sampling in the grounded theory methodology. During the entire data collection process, we intended to find health care professionals working in health care institutions, but they were often too over-worked or not motivated to participate. Due to the difficulties in recruiting health care professionals, a questionnaire was sent to a wide variety of primary, secondary and tertiary clinics and hospitals in Serbia. However only a small number responded, and it became clear that there was a need for better collaboration, networking, and face-to-face communication to recruit these respondents. Gifts or other honorariums could have been provided to motivate participation. Time and budget constraints, however, made these initiatives unrealistic. Other researchers [92,93] have also acknowledged the limitations when recruiting participants for qualitative research. Some initiatives could be supported for non-English speakers, forming collaborations to improve access to participants and reducing the participation burden. The present study considered several of these initiatives, although there was a need for broader and more comprehensive collaboration with relevant institutions to potentially have higher respondents’ participation.

When recruiting migrants, it was necessary to retrieve approvals for interviewing, which slowed the process. Only limited access was given to the migrant respondents and the asylum centre chose them on our behalf. The interviews were also under time constraints, which contradicts the time-consuming nature of qualitative research methodology [91]. Furthermore, we interviewed only those that were able to undergo an interview and thus not the migrants that were under greater physical or psychological pressure. Researchers [93,94] find that there may be several limitations to the willingness of migrants’ participation in research, namely their gender, religious beliefs, language barriers, educational level or fear of changes in their asylum process. A successful approach to recruit migrants may be via their own network [93]. We were thus present at their local hubs and the areas that they socialized in, to invite them to participate. If choosing to participate, migrants were offered gratitude gifts. However, as undocumented or moving migrants in vulnerable situations, they did not have the motivation to participate. Only migrants in the local asylum camp were interviewed.

Generally, the recruitment of participants was a long process. Sampling bias may be present in the study, potentially resulting in missing information and perspectives in the data.

In order to address potential bias in the analysis and coding of data [95,96], we conducted intercoder reliability (ICR) (for the present study, only one coder did the analysis). The ICR test was done using NVivo software which resulted in a Kappa’s coefficient (K), ranging from below 0 (no agreement) to 1 (total agreement). For the present ICR test, results below 0.41 were regarded as poor, whereas values from 0.60 were satisfactory or solid agreements and values above 0.80 were nearly perfect agreement [95,97]. Two researchers conducted the test on 10% of the data and initially met to discuss and agree upon the pre-selected categories to be coded that were selected by the main researcher [98]. After the first coding of the categories, the results were discussed, and the codes were revised to reach a higher level of agreement and reduce misunderstandings in categorization. A final test was done with the resulting mean Cohens Kappa 0.718, showing a high agreement level, thus indicating that the coding was consistent.

## 5. Conclusions

We sought to uncover the process inherent in paying informally in Serbia. In this regard, the present study concluded that migrants and local residents had different pathways to and though the Serbian health care system. The newly arrived migrants were given special attention by authorities, due to the humanitarian situation. Therefore, differences were seen in the organization of health care between the two groups and thus also the ability or need to pay informally. The newly arrived migrants neither experienced the same barriers as other vulnerable groups, such as the financial barriers, waiting times and physical distance. This situation illustrates the ability and willingness to organize services in a manner that reduces unequal access to health care. Overall, mainly vulnerable groups faced obstacles when accessing health care in Serbia and it may therefore be possible to learn from the structural factors seen in addressing the access to care for newly arrived migrants, such as the use of intercultural mediators. The intercultural mediator’s role in the process to paying informally, however, needs more investigation. However, being a country with social and financial difficulties, one of the main perceived reasons for paying informally, and thus poor health system governance, was the lack of health care resources. Consequently, poor working conditions, low salaries of doctors or lacking equipment or drugs in the institutions were seen. The access to care of vulnerable groups, therefore, seems to be a consequence that stems from a wider systems failure and tackling this issue demands actions from several areas of the health care system.

Gift giving was identified as a duplicitous phenomenon, where some found it unacceptable and illegal while also being perceived as a value-laden practice that was necessary. Doctors were thus perceived to be very important in society and there was a need to motivate them with gifts and money. Such beliefs are discussed in numerous literature and illustrate the difficulty when attempting to eradicate the practice. Informal patient payments appeared pervasive and rooted in Serbian society and were, to a certain extent, legalized in the newest health care legislation. Such legalization of informal patient payments was perhaps based on an acceptance of important national-cultural aspects to be taken into consideration. Informal patient payments are, however, typically rooted in poorly functioning health care systems, and it is therefore highly important to not confuse cultural beliefs with forced spending on health care. Private payments could be involuntary from the patient’s side.

The present study found a commonality of paying informally or using private care in Serbia, together with the identification of high level of out-of-pocket payments. Despite the majority of the population being covered by the national health insurance, the high level of out-of-pocket payments showed the staggering barriers to health care that are present in Serbia. Such private out-of-pocket spending for health care should be reduced in order not to push people further into poverty.
